# Randomized trial of intravenous versus bidirectional chemotherapy after cytoreductive surgery for malignant peritoneal mesothelioma

**DOI:** 10.1097/SP9.0000000000000010

**Published:** 2023-09-26

**Authors:** Paul H. Sugarbaker

**Affiliations:** Program in Peritoneal Surface Malignancy, Washington Cancer Institute, Washington, DC, USA

**Keywords:** HIPEC, pemetrexed, peritoneal metastases, cisplatin, intraperitoneal chemotherapy, peritonectomy, cytoreductive surgery

## Abstract

**Background:**

Malignant peritoneal mesothelioma (MPM) is a rare disease that progresses within the peritoneal cavity and only disseminates to systemic sites in the terminal months of the disease. For this malignancy, there are several regimens of chemotherapy that have been accepted as standard, principally intraperitoneal chemotherapy (IPC) and intravenous chemotherapy (IVC); however, there is no standardized method of treatment. Selected patients with MPM who are amenable to cytoreductive surgery (CRS) and are fit for surgery typically undergo resection with hyperthermic intraperitoneal chemotherapy (HIPEC). Though individual toxicity and efficacy studies of IPC plus IVC (bidirectional) and IVC chemotherapy for MPM have been conducted, a prospective randomized clinical trial has not been performed for this disease.

**Materials and methods:**

The study objective is to compare the efficacy and toxicity of normothermic bidirectional (IPC/IVC) chemotherapy versus IVC after CRS and HIPEC for epithelial MPM. The patient population are those individuals undergoing CRS for MPM. Exclusion criteria include previous therapy form mesothelioma. The study design is a randomized, nonblinded, phase II clinical trial comparing multicycle IVC with cisplatin (CDDP) and pemetrexed (PMTX) versus multicycle bidirectional chemotherapy with IVC CDDP and IPC PMTX after optimal CRS and HIPEC with CDDP and doxorubicin. The primary endpoint is 2-year disease-free survival. The secondary endpoint is 30-day post-treatment morbidity. The primary objective is to compare the 2-year rates of disease-free survival in the two treatment arms. The secondary objective is to compare the toxicity of each treatment.

**Dissemination:**

The prospective randomized trial provides not only a standardized approach to treatment but also a path forward to optimize the survival of patients with MPM. In addition, any increase or decrease in the adverse events associated with PMTX administered as IPC will be demonstrated. Because MPM is a rare disease a multi-institutional implementation of the protocol is required.

## Background and rationale

### The natural history of MPM

Highlights

- Malignant peritoneal mesothelioma is a disease characterized by progression within the peritoneal cavity.
- Cytoreductive surgery with hyperthermic intraperitoneal chemotherapy has increased 5-year survival to 50%.
- Pemetrexed (PMTX) is a chemotherapy agent especially well-suited for intraperitoneal administration.
- Preliminary data shows that long-term intravenous cisplatin plus intraperitoneal PMTX prolongs 5-year survival to 75% when added to cytoreductive surgery and hyperthermic intraperitoneal chemotherapy.
- A prospective randomized trial of intravenous cisplatin plus PMTX versus intravenous cisplatin plus intraperitoneal PMTX for six cycles is proposed.

Malignant peritoneal mesothelioma (MPM) of the epithelial type is a rare disease with ~300 new cases per year in the United States. The natural history of MPM is progression predominantly on peritoneal surfaces and very rarely outside the abdomen. Unlike patients with pleural mesothelioma, many patients with MPM have no known history of asbestos exposure. MPM is characterized by the peritoneal dissemination of metastases with mucinous or serous ascites early in the course of the disease. MPM collects in the omentum as well as dependent portions of the abdomen, such as the pelvis, retrohepatic space, and paracolic gutters. The small and large intestines may be extensively involved. However, MPM does not commonly involve lymph channels, venules, or distant solid organ parenchyma, for example, the liver or lungs. Most cases of MPM-associated morbidity and mortality result from intestinal obstruction and malnutrition. Prior to the 1980s, MPM was managed with systemic chemotherapy, and surgical procedures were reserved for palliation of bowel obstruction and ascites. Initial reports of systemic chemotherapy for MPM showed low response rates and poor long-term survival. However, more recent reports of findings in patients eligible for cytoreductive surgery (CRS) showed higher response rates to systemic cisplatin and pemetrexed (PMTX)^1^ and a median progression-free survival of approximately 18 months^2,3^.

### CRS plus hyperthermic intraperitoneal chemotherapy (HIPEC) for MPM

Since the 1980s, CRS plus HIPEC procedures have been utilized to eliminate all tumor visible to the surgeon’s unaided eye in several types of cancer, with the expectation that survival will be prolonged^4^. CRS that achieves optimal excision of peritoneal disease (with residual nodules less than 2.5 mm) in highly selected patients has been associated with survival that is significantly longer than that of patients undergoing suboptimal excision. A medial survival of ~5 years has been reported in retrospective series of CRS plus HIPEC patients, compared to a 1-year median survival for historical control MPM patients who did not have CRS^5,6^. However, small-volume gross or microscopic disease often remains after CRS. Even though HIPEC was used, a majority of patients have abdominal and pelvic progression of disease after surgery. The high prevalence of residual/recurrent disease led investigators to evaluate protocols that would deliver long-term intraperitoneal chemotherapy (IPC) to the anticipated site of recurrence. This regional approach can deliver high concentrations of drug which can penetrate less than 1 mm of peritoneal tissue. Retrospective analysis of outcomes in patients who underwent CRS and HIPEC indicates that this approach may offer longer survival than systemic chemotherapy alone and appears commonly used in MPM patients who are appropriate candidates for surgery^6^.

### HIPEC for MPM

HIPEC has been developed in response to challenges associated with the tolerability and logistics of multicycle long-term IPC. Initially, a normothermic solution of chemotherapy was used. Subsequently, intraoperative intraperitoneal hyperthermic chemotherapy (shown to be technically more acceptable) was integrated into the CRS treatment paradigm of peritoneal metastases in order to increase tissue penetration and cytotoxicity of the antineoplastic agent delivered^4^. Hyperthermia itself may be cytotoxic to tumor cells, based on established mechanisms involving inhibition of nuclear matrix-mediated functions essential to DNA replication, transcription, and repair^7,8^. However, it is the combined antitumor effect of heat and IPC that serves as the basis for the widely practiced HIPEC for peritoneal carcinomatosis^4^. The advantages of HIPEC are that (i) it is given before postoperative adhesions develop, (ii) hyperthermia may have a tumoricidal effect^9^, and (iii) hyperthermia may potentiate the cytotoxic effect of selected chemotherapy agents, such as cisplatin and doxorubicin, the drugs most commonly used in HIPEC^10–12^. The HIPEC approach has been further supported by data from a randomized trial in patients with peritoneal metastases of colorectal or appendiceal origin^13^. That study demonstrated a survival benefit associated with CRS-HIPEC compared to systemic chemotherapy and palliative surgery when necessary (median survival 22.3 vs. 12.6, *P*=0.032).

CRS-HIPEC has become the most widely used treatment for resectable MPM^14^. However, despite routine use of CRS-HIPEC, recurrence, most frequently peritoneal, typically happens within 12–24 months. IPC and intravenous chemotherapy (IVC) have been used, though inconsistently, in the postoperative setting for patients with MPM. This consolidation approach after CRS-HIPEC has been associated with better long-term survival compared to historical controls^15,16^. However, the effectiveness of HIPEC has not been prospectively evaluated, nor have the relative benefits of long-term postoperative IPC been directly compared with those of postoperative IVC. IVC is an appealing approach after CRS, as it is widely accessible to patients. In contrast, IPC requires a higher level of expertise at select specialty centers but can achieve higher concentrations of chemotherapy on the peritoneal surfaces, where the risk of relapse is highest. The primary aim of the proposed randomized trial is to compare multicycle IVC using cisplatin plus PMTX versus multicycle bidirectional chemotherapy with IVC cisplatin and IPC PMTX for efficacy and toxicity in patients who have undergone CRS-HIPEC.

### IPC for MPM

Over the past three decades, IPC has been used for peritoneal metastases of various primary tumors, but no randomized trials have been conducted for MPM^17^. Initially, IPC was delivered via an intraperitoneal catheter placed at the time of cytoreduction^18,19^. This approach allowed high doses of cytotoxic chemotherapy to be delivered intraperitoneally with minimal systemic toxicity^4^. Although reports of outcomes after IPC were promising, concerns remained that the perfusion of peritoneal surfaces may not be uniform, because of the fibrotic reaction to surgical trauma, which can trap cancer cells. Theoretically, multiple cycles of IPC may allow for a greater cumulative tumor cell kill than a single cycle. However, IPC has proven to be difficult for some patients to tolerate due to pain, distension, and nausea^20^. Furthermore, giving multiple outpatient cycles of chemotherapy is logistically challenging for many patients, who may need to travel long distances to specialty centers to receive treatment for this rare condition. Because of these considerations and the lack of prospective data, IPC has not been practiced widely in potentially eligible patients. Nevertheless, IPC is utilized at centers that have the infrastructure to support its use^15^. In randomized trials, multicycle IPC has been demonstrated to be superior to postoperative IVC alone after CRS for ovarian cancer^21^. Additionally, the findings of a randomized clinical trial in patients with metastatic colon cancer suggest that combination of CRS and IPC is associated with longer overall survival than best systemic chemotherapy alone (HR 0.51, 95% CI: 0.27–0.96)^22^. Similarly, a retrospective study of 129 patients who underwent CRS for MPM at a single center reported that patients who received IPC had longer overall survival on average (HR 0.29, 95% CI: 0.12–0.67) than patients who did not receive IPC^15,16^.

### Pharmacologic rationale for IPC with PMTX

When PMTX was delivered intraperitoneally there is a delayed clearance of chemotherapy from the peritoneal fluid. The mean half-life of PMTX in the peritoneal fluid was 127 min. There was a slow increase in plasma concentration with a mean peak level occurring at 60 min followed by a gradual decrease during the next 30 min and then a stabilization until the end of the experiment. The AUC ratio of peritoneal fluid to plasma was 40.8. In the 6 h experiment PMTX concentration in the peritoneal fluid gradually decreased with 75% of the drug being absorbed over 6 h^23^.

There is a significant increase (*P*<0.0001) in the AUC for peritoneal fluid concentrations after intraperitoneal administration. The AUC for peritoneal fluid PMTX represents a 20-fold increase in exposure for tissues at peritoneal surfaces with intraperitoneal administration. The highest tissue concentration of PMTX was in the kidney (*P*=0.0122). In the other tissues, except for the liver, intraperitoneal administration exhibited higher PMTX concentrations that the intravenous administration including tissues from the abdominal wall, diaphragm, and mesenteric lymph nodes. These differences were statistically significant for the mesenteric nodes and the abdominal wall (*P*=0.0036 and 0.0017)^23^.

Another characteristic of PMTX that makes it a candidate for multicycle IPC is the lack of pain upon instillation and the absence of peritoneal sclerosis. It has been used in prior protocols with multiple intraperitoneal instillations. Even after many cycles of IPC administration, catheter access was unimpaired. Long-term follow-up of patients treated with multicycle PMTX have no digestive complaints or nutritional problems^15^. PMTX is one of the few chemotherapy agents especially well-suited for long-term IPC treatment.

## Overview of study design/intervention

### Design

**Figure 1:**
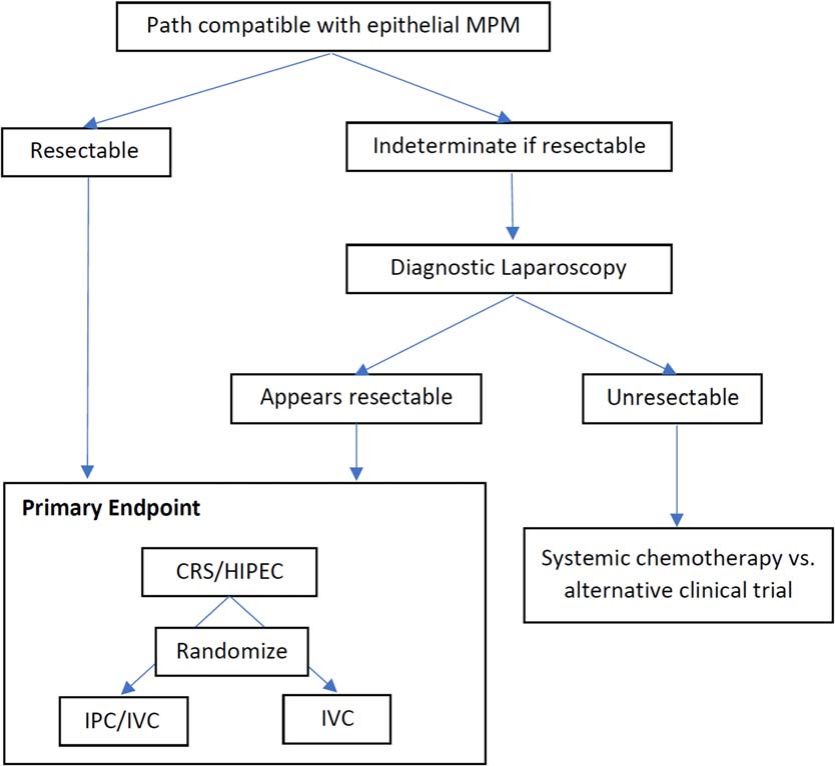
Diagram of prospective randomized trial of long-term intravenous cisplatin and pemetrexed versus bidirectional treatment with intravenous cisplatin and intraperitoneal pemetrexed.

This is a multicenter randomized, nonblinded, phase II clinical trial of the relative efficacy of the two existing postoperative chemotherapy regimens for MPM. The target population for this study is patients undergoing CRS and HIPEC for MPM. It will compare multicycle IVC with CDDP/PMTX and multicycle IPC/IVC with IVC CDDP and IPC PMTX after optimal or near-optimal (nodules no larger than 2.5 mm) CRS followed by HIPEC with CDDP plus doxorubicin. Disease-free survival and the toxicity of treatment will be measured (Fig. 1).

### Intervention

CRS of all affected abdominal surfaces will be performed in the usual fashion. This will include, when necessary, excision of prior abdominal incision, peritonectomy, bowel resection, oophorectomy, hysterectomy, omentectomy, splenectomy, and cholecystectomy. After the surgeon determines surgical cytoreduction is complete, defined as no residual peritoneal tumor nodules greater than 2.5 mm, intraoperative treatment with HIPEC will take place. HIPEC is by closed-abdomen or open-abdomen technique, per the surgeon’s discretion, for 90 min in the hyperthermic phase (41.5–42.5°C). The dose of CDDP is 50 mg/m^2^ and the dose of doxorubicin is 15 mg/m^2^. After completion of HIPEC, the residual chemotherapy solution is removed. After irrigation is complete, the abdomen and pelvis will be examined. Any intestinal anastomoses will be performed as needed. During or immediately after HIPEC, the patient will be randomized to IPC/IVC or IVC. Fascia and skin will be closed in a standard fashion.

### IVC arm

PMTX at 500 mg/m^2^ and cisplatin at 75 mg/m^2^ will be administered intravenously for a total of 6 cycles every 3 weeks. An intraperitoneal port will not be placed in patients receiving IV chemotherapy. The peritoneal access would carry a potential adverse event but would not be of any possible benefit to the patient.

### IPC/IVC arm

After completion of HIPEC, an intraperitoneal catheter or an intraperitoneal port will be inserted through the abdominal wall^24^. At ~4 weeks after CRS and HIPEC when the patient’s condition is considered stable by the physicians, bidirectional chemotherapy will be administered. PMTX at 500 mg/m^2^ given intraperitoneally in 1 l of peritoneal dialysis solution (1.5% dextrose) will be administered as rapidly as tolerated by the patient. Only after the IPC administration is complete, cisplatin at 75 mg/m^2^ will be administered intravenously. The delay in IV cisplatin administered is to allow the artificial ascites from PMTX to maximize. This facilitates the distribution of bloodborne cisplatin into the peritoneal cavity. This treatment is for 6 cycles every 3 weeks.

The rationale behind both IPC and IVC is that small-volume residual disease persists even after optimal CRS plus HIPEC, as evidenced by the majority of these patients who experience recurrence in the peritoneal cavity. If the patient has not received IPC/IVC or IVC within 8 weeks of CRS, the clinician is to determine if adjuvant chemotherapy will be administered. Regardless, the patient will remain in the study and will be included in the analysis, based on the intention to treat.

## Therapeutic/diagnostic agents

### Cisplatin

Cisplatin is commercially available in multidose vials of 50 or 100 mg. It is prepared as a sterile aqueous solution containing 1 mg of cisplatin and 9 mg or sodium chloride per ml. Vials are stored at 15–25°C (59–77°F). Do not refrigerate. Protect the unopened container from light. Store in a carton until the contents are used. The cisplatin remaining in the amber vial following initial entry is stable for 28 days if protected from light or for 7 days under fluorescent room light. Cisplatin can be administered intraperitoneally or intravenously.

NOTE: Aluminum reacts with cisplatin, causing precipitation formation and loss of potency; therefore, needle or intravenous sets containing aluminum parts that may come in contact with the drug must not be used for the preparation or administration of cisplatin.

### Doxorubicin

Doxorubicin is commercially available in multidose vials of 10, 20, 50, 100, or 150 mg. For preparation, add doxorubicin to a sterile solution or 5% dextrose and water for injection into the chemotherapy solution. Store vials at room temperature. Vials reconstituted to 2 mg/ml are stable for 7 days at room temperature. Doxorubicin can be administered intraperitoneally or intravenously.

### PMTX

PMTX (Alimta, Eli Lilly) is a commercially available medication with a complete description of the drug, clinical pharmacology, contraindications, warnings, precautions, and adverse reactions available in the package insert. The freeze-dried product is composed of PMTX and mannitol at a 1:1 ratio. Sodium hydroxide and/or hydrochloric acid solution may be added during processing to adjust the pH. Two vial sizes are available: 100 and 500 mg. Each vial contains the equivalent of 102 or 510 mg of PMTX. The vials contain 2% excess to facilitate withdrawal of the label amount from each 100 mg or 500 mg vial. The drug product is stable when stored at room temperature. Reconstituting the 100 mg vial contents with a 2 ml to 10 ml sodium chloride injection gives a clear solution with a concentration of 10 to 50 mg/ml. The reconstituted formulation is in the pH range of 6.8 to 7.5 and has been shown to be chemically stable for 72 h at refrigerated or room temperature. Vial contents should be inspected for particulate matter before and after the drug product is withdrawn from a vial into a syringe. The concentrated solution cannot be administered without further dilution. For purposes of clinical administration, an appropriate quantity of the contents of the reconstituted vials must be further diluted as per institutional guidelines. After dilution, the shelf life of the product is 24 h at room temperature. PMTX is not sensitive to light. Vials of PMTX contain no antimicrobial preservative, and they are single-use vials. Any unused portion of a vial may not be stored for future use, and unused portions of product in a vial must be discarded. PMTX is incompatible in solution with acidic medications or media and must not be mixed or administered simultaneously through the same infusion line. The infusion line should be flushed prior to the administration of any concomitant medication. PMTX is compatible with standard PVC administration sets and any IV solution bags. PMTX can be administered intraperitoneally or intravenously. Folic acid and vitamin B12 should be administered as per standard institutional practices 2 weeks before and continuously during treatment.

### Carboplatin (if cisplatin is contraindicated or not tolerated)

Carboplatin is available as a 10 mg/ml solution. When prepared as directed, carboplatin solutions are stable for 8 h at room temperature. Since no antibacterial preservative is contained in the formulation, it is recommended that carboplatin solutions be discarded 8 h after dilution. Carboplatin can be reconstituted with either sterile water for injection USP, 5% dextrose in water, or 0.9% sodium chloride injection USP in a vial strength/diluent volume ratio of 50 mg/5 ml, 150 mg/15 ml, or 450 mg/45 ml to produce a carboplatin concentration of 10 mg/ml. Reconstituted solutions can be further diluted in 5% dextrose in water or 0.9% sodium chloride for IV infusion. Unopened vials of carboplatin are stable to the date indicated on the package when stored at a controlled room temperature and protected from light. Carboplatin will be administered according to institutional guidelines as a 30 min infusion (standard protocol) or a 3 h infusion (standard desensitization protocol). Complete details are listed in the package insert. Aluminum reacts with carboplatin, causing precipitation and loss of potency; therefore, needle or intravenous sets containing aluminum parts that may come in contact with the drug must not be used for the preparation or administration of carboplatin.

## Criteria for subject eligibility

4.1. The subject inclusion criteria are shown in Table 1.

**Table 1:** Subject inclusion criteria.

Patient age 18 years or older, both sexes.
Clinical diagnosis of epithelial type MPM.
Patient must be planning to undergo complete cytoreduction of all peritoneal disease followed by HIPEC.
ECOG performance status of ≤1.
Hematology: ANC ≥1500/µl.
Platelets: >75 000/µl.
Adequate renal function: creatinine <1.5× the upper limit of normal (ULN) or calculated creatinine clearance of ≥50 ml/min.
Adequate hepatic function: bilirubin <1.5 mg/dl (except in patients with Gilbert’s syndrome, who must have a total bilirubin <3.0 mg/dl).
Women of childbearing potential with a negative pregnancy test result (urine or blood) who agree to use an effective contraceptive method. Reliable contraception should be used from trial screening and must be continued throughout the study. A woman of childbearing potential is defined as one who is biologically capable of becoming pregnant.
A man participating in this study must agree to utilize a reliable barrier form of contraception for the duration of the study.
Signed and dated written informed consent to participate in this clinical trial must be obtained prior to any study procedure.

. HIPEC, hyperthermic intraperitoneal chemotherapy; MPM, Malignant peritoneal mesothelioma

4.2. The subject exclusion criteria are shown in Table 2.

**Table 2:** Subject exclusion criteria.

Subjects who have previously undergone intraperitoneal chemotherapy or systemic chemotherapy for MPM.
Subjects who have previously received platinum-containing chemotherapy regimens.
Subjects with preoperative or intraoperative biopsy consistent with sarcomatoid MPM, biphasic MPM, well-differentiated papillary mesothelioma, or benign multicystic mesothelioma.
Other prior malignancies, except for cured nonmelanoma skin cancer, curatively treated *in situ* carcinoma of the cervix, adequately treated malignancies for which there has been no evidence of activity for more than 3 years, or indolent tumors for which observation over 2 years is a reasonable option.
High suspicion for extra-abdominal metastases.
Women who are pregnant or lactating.
Subjects with a condition which may interfere with their ability to understand the requirements of the study.
Active coronary artery disease (defined as unstable angina or a positive cardiac stress test). Subjects with a history of coronary artery disease may be included if they have had a normal stress test within 60 days of enrollment or are determined by a cardiologist to be of acceptable perioperative risk.
Uncontrolled hypertension defined as >140/90 and not cleared for surgery at the time of consent.
New York Heart Association (NYHA) Class II or higher congestive heart failure; restrictive or obstructive pulmonary disease that would limit study compliance or place the patient at unacceptable risk for participation in the study.
History of cerebrovascular disease that would limit study compliance or place the patient at unacceptable risk for participation in the study.
Subjects with other concurrent severe medical problems unrelated to the malignancy that would significantly limit full compliance with the study or place them at unacceptable risk for participation in the study.
Patients with known cisplatin, carboplatin, or pemetrexed allergy.
Evidence of extensive intraperitoneal adhesions at the time of surgery which prohibits intraperitoneal therapy, as determined by the operating surgeon.
Any condition that would preclude the ability to deliver appropriate IPC.
Use of an oral medication, lacking a suitable nonoral substitute, that if held for up to 10 days would be felt an unacceptable risk by the investigator.
Life expectancy <12 weeks.

IPC, intraperitoneal chemotherapy.

## Recruitment plan

Potential research subjects will be identified by a member of the patient’s treatment team, the protocol investigator, or the research team at the participating institution. If the investigator is a member of the treatment team, he/she will screen the patient’s medical records for suitable research study participants and discuss with the patient their potential for enrolling in the research study.

The principal investigator may also screen the medical records of patients with whom they do not have a treatment relationship for the limited purpose of identifying patients who would be eligible to enroll in the study and to record appropriate contact information in order to approach these patients regarding the possibility of enrolling in the study.

This study will be available to all eligible patients, regardless of race, sex, or ethnic origin. The processes for minorities and women are the same as those for our general patient population. In addition, we will translate the informed consent form into additional languages for the non-English-speaking patient population when necessary.

There is an anticipated accrual of 23 patients per year. Accrual rates are expected to vary across sites based on location and available staff support. Patients will be screened and enrolled at the participating MPM site. It is required that patients have all of their treatment at the primary MPM enrolling site. However, they may receive some of their follow-up evaluations at local sites.

## Pretreatment evaluation

The pretreatment evaluation is shown in Table 3.

**Table 3:** Pretreatment evaluation.

Obtain a signed informed consent.
Obtain a complete medical history and history of anti-cancer therapy, date of onset, and initial status, as well as previous treatments and concomitant medications.
Obtain a CT scan of the chest, abdomen and pelvis within 60 days of surgery. MRI of the abdomen and pelvis with contrast may be used if CT results are inconclusive or if CT contrast is contraindicated. A noncontrast CT of the chest is required in situations when an MRI of the abdomen and pelvis is performed. PET/CT is an acceptable alternative.
Perform a complete physical examination: vital signs (blood pressure and heart rate), weight, height, and ECOG performance status.
Obtain an ECG.
Laboratory testing, including:
Chemistry.
Complete differential blood cell count (CBC), PT, PTT.
Pregnancy test for subjects of childbearing potential.
Review of each eligible patient by the MPM site principal investigator or MPM site co-principal investigator to ensure that eligible patients meet study criteria and are appropriate surgical candidates.
Screening to be completed within 30 days of study day 1.

## Treatment/intervention plan

- First, patients will be stratified by the initial intraoperative peritoneal carcinomatosis index (PCI) at the time of CRS, the completeness of cytoreduction (CCR), and sex.
  1. Estimated PCI ≤12 (low PCI) versus PCI >12 (high PCI)
  2. CCR 0/1 (0 to ≤2.5 mm; optimal) versus CCR 2 (>2.5–≤ 5 mm; suboptimal)
  3. Female versus male
- Then, patients will be randomly assigned in the operating room, by envelope, to either IVC (group A) or IVC/IPC (group B) after the operating surgeon has initiated HIPEC.

Patients in group A will receive six cycles of IVC with CDDP/PMTX. Patients in group B will receive six cycles of IVC/IPC with CDDP/PMTX with PMTX given by intraperitoneal port or intraperitoneal catheter.

### Patient cohorts

There will be eight cohorts based on the three levels of stratification. The stratifications are shown in Table 4.

**Table 4:** Eight cohorts based on three levels of stratification.

1. Low PCI, optimal CCR, female.
2. Low PCI, optimal CCR, male.
3. Low PCI, suboptimal CCR, female.
4. Low PCI, suboptimal CCR, male.
5. High PCI, optimal CCR, female.
6. High PCI, optimal CCR, male.
7. High PCI, suboptimal CCR, female.
8. High PCI, suboptimal CCR, male.

### Drug administration

#### Group A: IVC with CDDP/PMTX

When the patient’s condition is considered stable by the treating physicians, the IVC will be started between 3 and 8 weeks post-CRS/HIPEC. PMTX at 500 mg/m^2^ and cisplatin at 75 mg/m^2^ (or carboplatin AUC 4–5, if cisplatin is contraindicated) will be administered intravenously for a total of six cycles, given every 3 weeks.

#### Group B: bidirectional IVC/IPC with CDDP IVC and PMTX IPC

After completion of HIPEC, an intraperitoneal catheter or an intraperitoneal catheter with a subcutaneous port will be inserted through the abdominal wall^24^. When the patient’s condition is considered stable by the treating physicians, the IVC/IPC will be administered starting between 3 and 8 weeks post-CRS/HIPEC. PMTX at 500 mg/m^2^ in 1000 ml of 1.5% dextrose peritoneal dialysis solution will be administered intraperitoneally for a total of six cycles, given every 3 weeks. Cisplatin at 75 mg/m^2^ will be administered intravenously after the instillation of intraperitoneal PMTX is complete. In the event that carboplatin must be used in either Group A or Group B, it will be administered as an intravenous infusion at a dose of 800 mg/m^2^ diluted in normal saline (~125–250 ml). The dose of carboplatin will be capped at the BSA 2.2 m^2^.

The dose, duration, and time of administration of therapy may be modified as clinically indicated. Antiemetic regimens are permitted and the addition of alternate or additional antiemetics may be used at the discretion of the clinical team.

Premedications and antiemetics for subsequent chemotherapy cycles will be administered per standard institutional guidelines.

Carboplatin/Cisplatin Hypersensitivity Reactions: In routine clinical practice, acquired carboplatin hypersensitivity reactions are seen. Patients with a history of prior carboplatin hypersensitivity will not be eligible for this protocol. If a patient develops carboplatin hypersensitivity during subsequent systemic chemotherapy, the treatment will be stopped and may be continued later at the discretion of the attending medical oncologist. A prophylactic desensitization infusion may be used during the IV carboplatin portion of the study.

### Postoperative care

In general, patients from both arms will be maintained and managed according to standard institutional criteria. The place for postanesthesia recovery will be decided upon on an individual basis. Thromboembolic prophylaxis will be maintained using institutional guidelines.

## Evaluation preoperatively and during treatment

The study calendar for the evaluation of patients preoperatively and during protocol treatments are shown in Table 5.

**Table 5:** The study calendar for evaluation of patients preoperatively and during protocol treatments.

Study calendar	Preoperative	OR	Postoperative weeks		Postoperative month (+/· 30 days)					
Time point			6 (3–8)	12 (10–14)	6	9	12	15	18	24
Eligibility/consent	X^1^									
EKG	X^1^									
Pregnancy test	x^1^									
Height	X^1^									
Weight	X^1^		X	X	X	X	X	X	X	
ECOG	X^1^		X	X	X	X	X	X	X	X
CT scan/MRl^2^	X^1^		X^5^		X	X	X	X	X	X
PT/INR	X^1^									
CBC with diff^3^	X^1^									
COMP^4^	X^1^		X	X	X					
Albumin^4^	X^1^			X	X		X		X	

1 Acceptable if within 30 days of surgery.

2 Preoperative CT or MRI is acceptable within 60 days of surgery.

3 WBC, RBC, HGB, HCT, MCV, MCH, MCHC, RDW, Platelets, Differential Type, Neutrophil, Lymph, Mono, Eos, Baso, Luc, Abs Neut, Abs Lymph, Abs Mono, Abs Eos, Abs Basa, and Abs Luc.

4 BUN, Creatinine, Sodium, Potassium, Chloride, CO_2_, Calcium, Glucose, Total Bilirubin, Total Protein, Albumin, Alkaline Phosphatase, AST, ALT, Anion Gap, EGFR African American, EGFR Non-African American.

5 Prior to initiation of postoperative chemotherapy.

## Toxicities/side effects

Adverse events will be graded using the NCI Common Terminology Criteria for Adverse Events-Version 4.0. A copy of the CTCAE version 4.0 can be downloaded from the CTEP home page (http://ctep.info.nih.gov).

### Cisplatin

Cumulative and delayed leukopenia, thrombocytopenia, renal toxicity, electrolyte abnormalities, ototoxicity, nausea, vomiting, mucositis, malaise, anorexia, alopecia, hypersensitivity, allergic reaction, change in blood pressure, and fatigue. Visual changes, venous thrombosis, and the development of leukemia are rare and serious toxicities.

### Doxorubicin

Cumulated and delayed leucopenia is dose limiting, also thrombocytopenia and anemia. The nadir for leucopenia is 10–14 days with recovery within 21 days. Nausea and vomiting, sometimes severe are dose-related and preventable with antiemetic prophylaxis. Anorexia, diarrhea, mucositis with ulceration and necrosis of the colon have been reported. Alopecia is usually total with a higher dose bolus intravenous injection. Doxorubicin is a desiccant and will produce fibrosis of the peritoneal space unless sufficiently diluted. Cardiomyopathy is related to a total cumulative dose greater than 550 mg/m^2^.

### PMTX

Leukopenia, thrombocytopenia, anemia, change in blood pressure, temporary abnormalities in liver function tests, nausea, vomiting, constipation or diarrhea, loss of appetite, and fatigue. Occasional side effects include allergic reactions, kidney damage, fever, weight loss, rash, skin changes, dehydration, electrolyte abnormalities, shortness of breath, mouth and throat sores, and peripheral edema. Rare side effects include serious bleeding due to low platelets, severe anemia, and kidney failure.

### Carboplatin

Myelosuppression, nausea, vomiting, peripheral neuropathy, ototoxicity, hepatic toxicity, electrolyte imbalances, hypomagnesemia, hypocalcemia, hyponatremia, fatigue, allergic reactions, and alopecia. Rare toxicities including ototoxicity, nephrotoxicity, edema, nervous system disorders (dizziness, blurred vision), hepatic toxicity, fever, weight loss, interstitial pneumonitis, hemolytic uremic syndrome, and uremia.

### Hyperthermia

Hyperthermia up to 43°C on the peritoneal tissue surface is not known to pose risks specific to this treatment. Temperatures will be continuously monitored to avoid exposing peritoneal surfaces to excessive temperature. Exceeding the goal temperature could lead to thermal bowel injury, which may predispose patients to a higher risk of anastomotic leak and ileus.

### Cytoreductive surgery

Surgical site infection, venous thromboembolism, hemorrhage, fluid shifts during resuscitation, bowel injury, and anastomotic failure are known concerns specific to the use of aggressive CRS and HIPEC. The morbidity of this operation has been associated with the grade of disease, extent of disease, and number of gastrointestinal anastomoses. This is true for most abdominal cancer surgeries.

### IP catheter failure

The IP catheter or IP port plus catheter may be replaced if necessary to administer the PMTX drug. The diagnosis of IP administration complications can be made with the assistance of fluoroscopic examination of the catheter or IP port plus catheter and contrast infusion into the peritoneal cavity. IP catheters may be removed after the last cycle of IP chemotherapy.

### Toxicity management

Every effort will be made to minimize the length of treatment interruptions. Aggressive supportive care measures such as antiemetics and hydration for patients with nausea and vomiting will be implemented.

## Criteria for therapeutic response/outcome assessment

The primary endpoint is disease recurrence. Documentation of tumor recurrence will be made based on surveillance CT scans at 3 monthly intervals as determined by the attending radiologist, with clinical correlation from the treating physician. All notable lesions identified at the first CT performed after surgery will be monitored for progression. Any peritoneal lesions seen on the first postoperative scan will be considered baseline. If they progress during chemotherapy, or on a subsequent scan, based on the clinical impression of the study physician, they will be considered an event. If they remain stable, that patient’s status will be considered NED (no evidence of disease). Patients with measurable disease on their first postoperative scans (3–8 weeks postoperatively, before initiation of postoperative chemotherapy) will be monitored for tumor progression at the 6, 9, 12, 15, 18, and 24 months time points (+/- 30 days). Also, patients with no evidence of disease on their first postoperative scan will also be followed at the 6, 9, 12, 15, 18, and 24 months time points (+-- 30 days), and the attending physician will review and confirm that the patient remains NED. Recurrence may also be determined pathologically. Though no postoperative biopsies will be required by protocol, an incidental, pathological confirmation of a clinically evident recurrence will be considered an event, regardless of scans.

The secondary endpoint is treatment toxicity. We will evaluate toxicity up to 30 days after completion of on-protocol therapy for any clinically significant Grade 3–5 complications. All patients included in the study will be assessed for response to treatment, even if there are major protocol treatment deviations or if they are ineligible.

Each patient will be assigned one of the following categories: (1) NED, (2) progressive disease, (3) early death from malignant disease, (4) early death from toxicity, (5) early death because of other cause, or (6) unknown (not assessable, insufficient data).

All of the patients who met the eligibility criteria will be included in the main analysis of the response rate. Patients in response categories 3–5 will be considered as failing to respond to treatment (disease progression). Thus, an incorrect treatment schedule or drug administration does not result in exclusion from the analysis of the response rate. Patients in category 6 will be censored at the time of the last follow-up.

## Criteria for removal from the study

A patient has the right to withdraw from the study at any time for any reason without prejudice to his/her future medical care by the physician or at the institution. The investigators also have the right to withdraw patients from the study (see below). Should a patient (or the patient’s legally authorized representative) decide to withdraw, all efforts should be made to complete and report the observations as thoroughly as possible. A final evaluation should be made at the time of the patient’s withdrawal. An explanation of why the patient is withdrawing, and an attempt is made to perform a follow-up evaluation. If a patient is found to be ineligible for the protocol as designated in the section on Criteria for Patient/Subject Eligibility (i.e. a change in diagnosis), the patient will be removed.

In addition, patients may be removed from the study if one or more of the following events occur:

- Significant noncompliance by the patient.
- Patient’s refusal to continue observations.
- Decision by the investigator that termination is in the patient’s best medical interest.
- Unrelated medical illness or complication.
- Loss to follow-up.

If a recurrence is discovered at one of the designated assessment time points, the patient will complete all remaining exams for that calendar visit. However, if recurrence is discovered at a nondesignated assessment time point, the patient will complete scheduled blood work at the subsequent calendar visit.

A patient who withdraws consent to participate in the study (i.e. patient choice) will be removed from the study.

Should he/she so desire, any patient withdrawing from the study will still undergo the surgical procedure, performed as originally scheduled.

## Biostatistics

This is a randomized, unblinded, multicenter Phase II clinical trial comparing efficacy and treatment toxicity between groups treated with IVC or IVC/IPC. Eligible patients will have completed an optimal surgical cytoreduction and HIPEC for MPM. Though individual toxicity and efficacy studies of IPC and IVC have been conducted, comparison trials of IVC versus IVC/IPC have not been completed. Response rates to IVC for MPM range from 30 to 40%; however, it is unknown if systemic chemotherapy offers a survival advantage after CRS. There is recent retrospective evidence that IPC is associated with better long-term survival compared to expectant management^15,16^. The secondary objective will be cumulative grade III and IV toxicity.

Currently, the collaborating sites estimate ~40 eligible patients per year. Based on current patient volume, we will need to randomize 53% of 120 patients seen over a 3-year period, for a rate of 1.78 patients per month. Sixty-four patients will be 1:1 randomized and stratified based on estimated volume of disease, CCR, and sex (see section 7, Table 4). This provides ~40 events with a median follow-up of 2 years, and 80% power to detect a 25% difference in the proportion of patients {50% vs. 75%, corresponding to a hazard ratio of 0.5) with disease progression or death within 2 years of CRS, assuming a log-rank test and a two-sided type I error of 20%.

We anticipate a less than or equal to 4% dropout rate. If a patient does dropout, he/she would be included in the analysis on the basis of intention to treat.

The provision that pathologically proven recurrence is counted even if there is no evident recurrence on imaging may lead to a bias in the comparison. If one of the two treatments is more likely to induce symptoms necessitating a workup, recurrence is more likely to be identified. To avoid this potential bias, for the purposes of analysis the next scheduled scan date will be used as the recurrence date for pathologically discovered recurrences. If no scan was scheduled at the time of pathological confirmation, the date of the next CT will be used for analysis.

Morbidity will be graded using the Common Terminology Criteria for Adverse Events (CTCAE) version 4.0. We will monitor mortality throughout the trial using statistical stopping rules. Specifically, we will stop accrual if there are two deaths within the first 15 patients, three deaths within the first 30 patients, or four deaths within the first 50 patients. If the true probability of death is 2.5%, the probability of early stopping is only 8%. This probability increases to 80% if the true mortality rate is 10%.

## Research participant registration and randomization procedures

### Research participant registration

Eligibility must be confirmed as defined in the section entitled Inclusion/Exclusion Criteria (Tables 1 and 2). Obtain informed consent, by following the procedures defined in the section entitled Informed Consent Procedures. During the registration process registering individuals will be required to complete a protocol-specific Eligibility Checklist. The individual signing the Eligibility Checklist is confirming whether or not the participant is eligible to enroll in the study. Study staff are responsible for ensuring that all institutional requirements necessary to enroll a participant to the study have been completed.

To complete registration and enroll a participant from another institution, the participating site must contact the study coordinator to notify him/her of the participant registration. The following documents must be sent to the study coordinator for each enrollment within 24 h of the informed consent being signed:

- The complete or partially completed eligibility checklist.
- The signed informed consent and HIPAA Authorization form.
- Supporting source documentation for eligibility requirements (e.g. laboratory results, pathology reports, radiology reports, MD notes, physical exam sheets, medical history, prior treatment records and EKG report).

### Randomization

Sixty-four patients will be randomized (1:1) to either arm A (IVC) or arm B (IVC/IPC) intraoperatively following CRS with HIPEC after all eligibility criteria are established. Randomization will be accomplished by the method of random permuted block, and patients will be stratified by the initial PCI (low PCI or high PCI), the CCR (optimal or suboptimal) and sex (male or female). Sealed, opaque, nonresealable envelopes containing the assignment form will be used. The Research Study Assistant will be responsible for executing the process for all sites. In patients randomized to arm B, a means for intraperitoneal access will occur prior to abdominal closure.

## Data and safety monitoring and protection of human subjects

The Data Safety and Monitoring (DSM) Plans were approved by the National Cancer Institute in September 2001. The plans address the new policies set forth by the NCI in the document entitled ʻPolicy of the National Cancer Institute for Data and Safety Monitoring of Clinical Trialsʼ, which can be found at http://cancertrials.nci.nih.gov/researchers/dsm/index.html.

There are several different mechanisms by which clinical trials are monitored for data safety and quality. There are institutional processes in place for quality assurance (e.g. protocol monitoring, compliance and data verification audits, therapeutic response, and staff education on clinical research quality assurance) and departmental procedures for quality control.

During the protocol development and review process, each protocol (e.g. in-house sponsored, industry sponsored, cooperative group, etc.) is assessed for its level of risk, and monitoring procedures are established at the time of protocol activation.

### Protection of human subjects

#### Benefits/risks

The potential benefits for subjects who participate in this study are derived from an intensive regimen of treatments that may maximize the chance for longer survival. The potential risks for subjects participating in this study would be the risk of hyperthermic tissue injury (not identified to date), toxicity from chemotherapy, and morbidity of surgery.

#### Toxicities/side effects

Leukopenia, thrombocytopenia, nausea, vomiting, fatigue, and electrolyte abnormalities are some of the most commonly reported adverse side effects. Such events are generally self-limited and respond to supportive care. There is a possibility that this regimen may increase the risk of postoperative complications. Such events will be treated appropriately as postoperative care requires.

#### Alternatives/options

The current treatment options for patients eligible for this study include CRS and HIPEC with or without IPC, palliative chemotherapy and/or surgery, a different clinical trial or the best supportive care. Whether or not a patient chooses to participate in this study will not affect the availability of standard, supportive, or other investigational treatment.

#### Financial costs/burdens

The interventions are widely used for MPM patients and are covered by most insurance plans.

#### Voluntary nature of the study

Participation in this study is entirely voluntary. The risks, benefits, toxicities/side effects, alternative options for treatment, financial costs/burdens and voluntary nature of the study will be explained to patients.

### Privacy

The Privacy Office may allow the use and disclosure of protected health information pursuant to a completed and signed Research Authorization form. The use and disclosure of protected health information will be limited to the individuals described in the Research Authorization form. A Research Authorization form must be completed by the Principal Investigator and approved by the IRB.

### Serious adverse event (SAE) reporting

An adverse event is considered serious if it results in any of the following outcomes:

- Death.
- A life-threatening adverse event.
- An adverse event that results in inpatient hospitalization or prolongation of existing hospitalization.
- A persistent or significant incapacity or substantial disruption of the ability to conduct normal life functions.
- A congenital anomaly/birth defect.
- Important Medical Events (IME) that may not result in death, be life-threatening, or require hospitalization may be considered serious when, based upon medical judgment, they may jeopardize the patient or subject and may require medical or surgical intervention to prevent one of the outcomes listed in this definition.

Note: Hospital admission for a planned procedure/disease treatment is not considered an SAE.

SAE reporting is required as soon as the participant signs consent. SAE reporting is required for 30 days after the participant’s last investigational treatment or intervention. Any events that occur after the 30-day period and that are at least possibly related to protocol treatment must be reported.

If an SAE requires submission to the IRB office, the SAE report must be sent to the IRB within five calendar days of the event. The IRB requires a SAE report be submitted electronically to the SAE Office.

The SAE report should contain the following information:

- Subject’s initials.
- Medical record number.
- Disease/histology (if applicable).
- Protocol number and title.

Data needing to be entered:

- The date the adverse event occurred.
- The adverse event.
- The grade of the event.
- Relationship of the adverse event to the treatment (drug, device, or intervention).
- If the AE was expected.
- The severity of the AE.
- The intervention.
- Detailed text that includes the following:

An explanation of how the AE was handled.
A description of the subject’s condition.
Indication if the subject remains on the study.

- If an amendment will need to be made to the protocol and/or consent form.
- If the SAE is an unanticipated problem.

The PI’s signature and the date it was signed are required on the completed report.

## Informed consent procedures

Before protocol-specified procedures are carried out, consenting professionals will explain full details of the protocol and study procedures as well as the risks involved to participants prior to their inclusion in the study. Participants will also be informed that they are free to withdraw from the study at any time. All participants must sign an IRB-approved consent form indicating their consent to participate. This consent form meets the requirements of the Code of Federal Regulations and the Institutional Review Board of this Center. The consent form will include the following:

1. The nature and objectives, potential risks, and benefits of the intended study.
2. The length of the study and the likely follow-up required.
3. Alternatives to the proposed study. (This will include available standard and investigational therapies. In addition, patients will be offered an option of supportive care for therapeutic studies.)
4. The name of the investigator(s) responsible for the protocol.
5. The right of the participant to accept or refuse study interventions/interactions and to withdraw from participation at any time.

Before any protocol-specific procedures can be carried out, the consenting professional will fully explain the aspects of patient privacy concerning research specific information. In addition to signing the IRB informed consent, all patients must agree to the Research Authorization component of the informed consent form.

Each participant and consenting professional will sign the consent form. The participant must receive a copy of the signed informed consent form.

## Ethical approval and consent to participate

The randomized trial is to be offered to a single as yet not identified institution. This institution will construct an appropriate consent with the ethics committee/institutional review board.

## Consent

The participating institution will construct an appropriate consent with the guidance of its ethics committee/institutional review board.

## Sources of funding

Administrative and secretarial support was provided by Foundation for Applied Research in Gastrointestinal Oncology (FARGO).

## Author contribution

The author has accepted responsibility for the entire content of this manuscript and approved its submission. P.H.S.: conceived and designed the study protocol. He provided the intellectual content and approved the final version for publication.

## Conflicts of interest disclosures

Author states no conflicts of interest.

## Research registration unique identifying number (UIN)

1. Name of the registry: not applicable.
2. Unique identifying number or registration ID: not applicable.
3. Hyperlink to your specific registration (must be publicly accessible and will be checked): not applicable.

## Guarantor

Paul H. Sugarbaker, MD.

## Data availability statement

Data sharing not applicable to this article as no datasets were generated or analyzed during the writing of the current study protocol.

## Provenance and peer review

Not an invited paper.
